# Evidence of telomere attrition and a potential role for DNA damage in systemic sclerosis

**DOI:** 10.1186/s12979-022-00263-2

**Published:** 2022-01-27

**Authors:** Alicia Usategui, Cristina Municio, Elena G. Arias-Salgado, María Martín, Beatriz Fernández-Varas, Manuel J. Del Rey, Patricia Carreira, Antonio González, Gabriel Criado, Rosario Perona, José L. Pablos

**Affiliations:** 1 Servicio de Reumatología, Instituto de Investigación Hospital 12 de Octubre, 28041 Madrid, Spain; 2 Servicio de Telomeropatías, Instituto de Investigaciones Biomédicas CSIC/UAM; CIBER enfermedades raras; Instituto de Investigación Hospital Universitario La Paz, Madrid, Spain; 3 Universidad Complutense de Madrid, Madrid, Spain; 4 Experimental and Observational Rheumatology, Instituto de Investigacion Sanitaria, Hospital Clínico Universitario de Santiago, Santiago de Compostela, Spain

**Keywords:** Systemic sclerosis, Telomere length, Fibroblast

## Abstract

**Background:**

To investigate the role of cell senescence in systemic sclerosis (SSc), we analyzed telomere shortening (TS) in SSc patients and the effect of targeting DNA damage in the bleomycin model of skin fibrosis.

**Results:**

Telomere length (TL) in blood leukocytes of 174 SSc patients and 68 healthy controls was measured by Southern blot, and we found shorter age-standardized TL in SSc patients compared to healthy controls. TL was shorter in SSc patients with ILD compared to those without ILD and in anti-topoisomerase I positive compared to anti-centromere positive patients. To analyze the potential role of DNA damage in skin fibrosis, we evaluated the effects of the DNA protective GSE4 peptide in the bleomycin mouse model of scleroderma and the fibrotic response of cultured human dermal fibroblasts. Administration of GSE4-nanoparticles attenuated bleomycin-induced skin fibrosis as measured by Masson’s staining of collagen and reduced *Acta2* and *Ctgf* mRNA expression, whereas transduction of dermal fibroblasts with a lentiviral GSE4 expression vector reduced *COL1A1, ACTA2* and *CTGF* gene expression after stimulation with bleomycin or TGF-β, in parallel to a reduction of the phospho-histone H2A.X marker of DNA damage.

**Conclusions:**

SSc is associated with TS, particularly in patients with lung disease or anti-topoisomerase I antibodies. Administration of GSE4 peptide attenuated experimental skin fibrosis and reduced fibroblast expression of profibrotic factors, supporting a role for oxidative DNA damage in scleroderma.

**Supplementary Information:**

The online version contains supplementary material available at 10.1186/s12979-022-00263-2.

## Background

The role of aging and cell senescence in fibrotic diseases is not clear since both pro- and anti-fibrotic cellular effects have been described. A consistent association between genetic or acquired telomere shortening (TS) and lung fibrosis has been identified in the last decades [1, 2]. Mutations of telomerase and shelterin genes are associated with TS and represent the most common genetic factor associated with familial idiopathic pulmonary fibrosis and less frequently with sporadic forms of adult-onset pulmonary fibrosis [3, 4]. TS has been mechanistically linked to lung fibrosis through the reduced capacity of respiratory epithelial cells with TS to recover from damage, resulting in enhanced connective tissue repair and fibrosis [5, 6]. Cell-specific telomerase deficiency in type II alveolar epithelial cells impairs stem cell function and induces cell senescence, and it is sufficient to induce lung fibrosis [7]. Fibroblast senescence is also recognized as a potential pro-fibrotic mechanism but somewhat paradoxically, telomerase activity in lung fibroblasts may contribute to the development of experimental lung fibrosis, [8–10]. Fibroblast senescence is characterized by the secretion of profibrotic mediators and restrains myofibroblast dedifferentiation and apoptosis, favoring their accumulation [10, 11]. Fibroblasts from systemic sclerosis (SSc) skin show replicative senescence and decreased autophagic capacity, and targeting either oxidative stress or cell senescence may ameliorate fibrogenesis [12–14]. However, impaired profibrotic signaling and extracellular matrix production is also one of the hallmarks of aged fibroblasts [15–17].

In SSc, the prevalence and clinical significance of TS is controversial. Telomere shortening (TS) was first described in 1996 in a small cohort of SSc patients, including limited and diffuse cutaneous SSc (lcSSc and dcSSc), and intriguingly, also in their family members [18]. This observation led to the hypothesis that environmental exposure to DNA damaging agents was the triggering factor, but the finding was not reproduced in another lcSSc cohort [19]. Shortened TL has recently been reported in some SSc subsets, such as those with lung involvement or in a small group with autoantibodies against telomeric proteins [20–22]. Differences between these studies may relate to the different sample sizes, normal TL reference, or to the use of different leukocyte samples and techniques to determine TL [23, 24].

Southern blot of terminal restriction fragments (TRF) provides an absolute and highly reproducible measure of TL [23]. We have taken advantage of this technique to determine TL in a large and prospectively characterized SSc cohort [25] and in healthy controls. We also examined the ability of the dyskerin derived GSE4 peptide, which protects DNA from oxidative damage and induces telomerase activity, to modify experimental skin fibrosis and to regulate profibrotic gene expression in cultured human fibroblasts [26–28].

## Results

### Telomere length in SSc patients and healthy controls

**Fig. 1:**
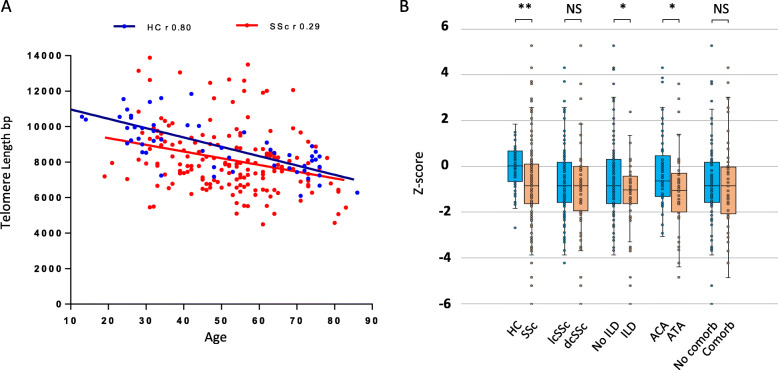
Telomere length in SSc patients and healthy controls. **A** TL in bp as determined in whole blood DNA by TRF Southern blot in healthy controls (blue, n 68) and SSc patients (red, n 174) plotted against age. r indicates the TL/age correlation coefficient by Spearman rank test, SSc *p* = 0.0001. HC *p* < 0.0001. **B** TL z-score of healthy controls, SSc patients, and SSc clinical and serological subsets. Median and IQR [25,75%] are represented. HC: healthy controls, lcSSc: limited cutaneous SSc, dcSSc: diffuse cutaneous SSc, ILD: Interstitial lung disease, ATA+: anti-topoisomerase-I positive, ACA+: anti-centromere protein B positive, Comorb.: Comorbid conditions, NS: Non significant, *p < 0.05, ** *p* < 0.0001

In both healthy controls (HC) and SSc groups, TL distribution showed a significant inverse correlation with age (Fig. 1A). A higher dispersion with a lower correlation coefficient were observed within the SSc group. SSc patients had a significantly decreased aged-adjusted z-score compared to HC (− 0.84 [− 1.15, − 0.62] vs − 0.00 [− 0.28, 0.31], *p* < 0.0001; Fig. 1B). Also, a higher proportion of patients with SSc showed a severely decreased TL, under the z-score 10th percentile, (40.8%) compared with HC (8.6%), *p* < 0.0001.

All clinical and serological SSc subsets had lower median TL compared to the HC group. The distribution of TL in the different SSc groups with different clinical and serological characteristics patients is shown in Table 1 and Fig. 1.

**Table 1:** Age adjusted TL in SSc patients and controls

	N	TL (z-score)^1^	p	<Percentile 10th n (%)	p
Healthy Controls	68	−0.00 [− 0.28, 0.31]	Ref	6 (9%)	Ref
SSc patients	174	−0.84 [−1.15, − 0.62]	< 0.0001	71 (41%)	< 0.0001
dcSSc	69	−0.84 [−1.93, 0.05]	< 0.0001	29 (42%)	< 0.0001
lcSSc	105	−0.85 [−1,57, 0,18]	< 0.0001	40 (38%)	0.0001
ILD	57	−1.03 [− 1.63, −0.42]	< 0.0001	24 (41%)	< 0.0001
No ILD	117	−0.83 [−1.62, 0.32]	0.004	45 (39%)	0.0001
ATA+	54	−1.04 [− 1.6, −0.03]	0.0002	22 (41%)	< 0.0001
ACA+	62	−0,63 [−1.30, 0.47]	0.025	20 (32%)	0.0008
Comorbidity^2^	115	−0.85 [−1.58, 0.18]	0.0001	44 (38%)	< 0.0001
No comorbidity	59	−0.84 [−2.06, − 0.02]	0.0003	27 (28%)	< 0.0001

^1^Median (IQR, 25–75%). ^2^ Comorbidity includes hypertension, hypercholesterolemia, diabetes, smoking or major adverse cardiovascular events (MACE). lcSSc: limited cutaneous SSc. dcSSc: diffuse cutaneous SSc. ILD: Interstitial lung disease. NS: Non significant. ATA+ (anti-topoisomerase-I positive), ACA+ (anti-centromeric protein B positive)

A lower TL was observed in the group of SSc patients with ILD compared to that without ILD (− 1.03 [− 1.63, − 0.42] vs − 0.83 [− 1.62, 0.32]; p 0.046). Also, patients with anti-topoisomerase-I autoantibodies (ATA) showed a lower median TL compared to patients with anti-centromere (anti-centromeric protein-B) autoantibodies (ACA) (− 1.04 [− 1.6, − 0.03] vs − 0,63 [− 1.30, 0.47]; p 0.035). Since ILD is strongly associated to ATA, we analyzed TL in patients with ILD with or without ATA. TL was similarly decreased in patients with ILD, regardless their ATA status (− 1.04 [− 1.63, − 0.54] vs − 0.83 [− 1,53,-0,17], *p* = 0.43).

We did not find differences in TL between lcSSc and dcSSc groups, nor by the presence or absence of previous comorbid conditions previously described associated with TS in the whole SSc group. Differences between SSc patients on different therapies, the presence of gastrointestinal, renal, cardiac involvement, or pulmonary arterial hypertension, were not detected (data not shown). No significant differences between the different SSc subsets regarding the proportion of severe TS (under percentile 10th) were observed. We did not find significant correlations between duration of the disease, extension of skin involvement measured as total skin score (TSS), nor lung fibrosis severity measured as forced vital capacity (FVC), and TL z-scores (data not shown).

### Effects of the GSE4 peptide on experimental skin fibrosis and human fibroblasts

GSE4 is a dyskerin related peptide that may induce telomerase activity and prevents telomere attrition induced by oxidative stress and DNA damage in different models of telomeropathies [26–28]. To analyze the potential effects of GSE4 on the development of skin fibrosis, we analyzed the effect of subcutaneous administration of GSE4-loaded nanoparticles in the bleomycin-induced model of scleroderma.

**Fig. 2:**
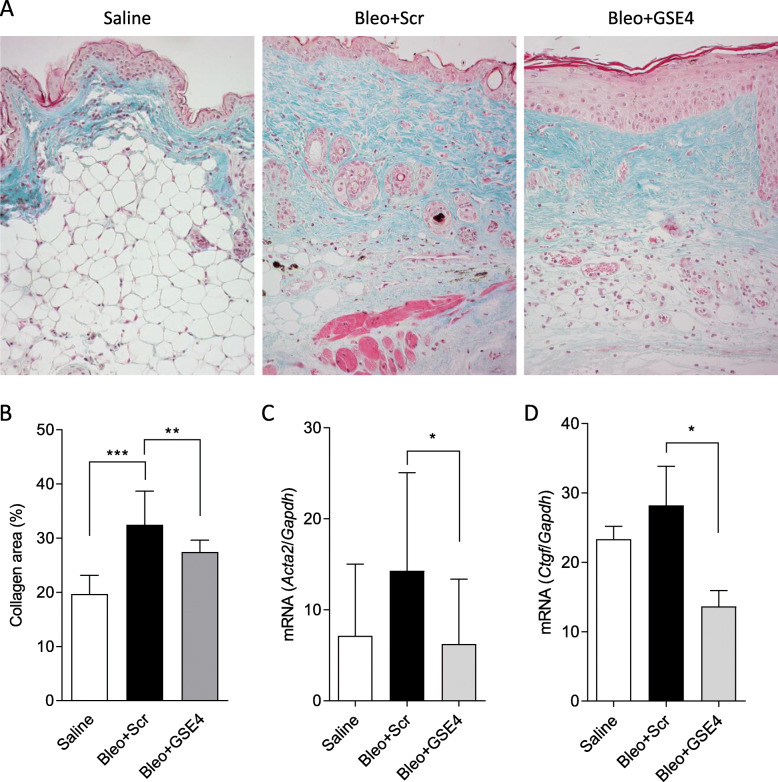
Effect of GSE4-nanoparticles on the development of bleomycin-induced skin fibrosis. C3H mice received daily subcutaneous injections of bleomycin, and were also injected with either scramble (Bleo + Scr) or GSE4 (Bleo + GSE4) nanoparticles every other day for 4 weeks. Control mice were daily injected with saline. **A** Representative image of Masson’s stained skin biopsy for each group. **B** Fibrosis was measured as the fold increase in the collagen Masson stained area adjusted to the normal area (saline group). Quantification of mRNA expression by quantitative RT-PCR of *Acta2* (**C**) *and Ctgf* (**D**). *p < 0.05, **P < 0.01, ***p < 0.001 (Median and IQR, Mann-Whitney test). Graphic shows data of 3 independent experiments with 10 mice per group. Bar 50 μm

In bleomycin injected mice, dermal fibrosis and an increased collagen-stained area compared to control, saline-injected mice, were observed (Fig. 2A). The fibrotic area, evaluated as the fractional Masson’s collagen-stained area of the dermis at 4 weeks, was significantly reduced in the group treated with GSE4-nanoparticles compared to the scramble peptide nanoparticles control group (Fig. 2B). Gene expression of additional markers of fibrosis such as the myofibroblast marker α-smooth muscle actin (*Acta2*) and the TGF-β inducible connective tissue growth factor (*Ctgf*) was also consistently reduced in the skin of the GSE4 treated group (Fig. 2C and D).

To examine the potential effects of GSE4 in human normal or SSc dermal fibroblasts, we treated cells with TGF-β or bleomycin, two pro-fibrotic stimuli associated with the production of reactive oxygen species (ROS) and DNA damage in fibroblasts [29–31]. Although some differences were observed in the individual responses of different fibroblast lines, both SSc and control fibroblasts groups responded to TGF-β or bleomycin by increasing the expression of profibrotic markers to a similar extent. Therefore, pooled data from SSc and normal fibroblasts on the effects of lentiviral expression of GSE4 and control peptides in fibroblasts are shown.

**Fig. 3:**
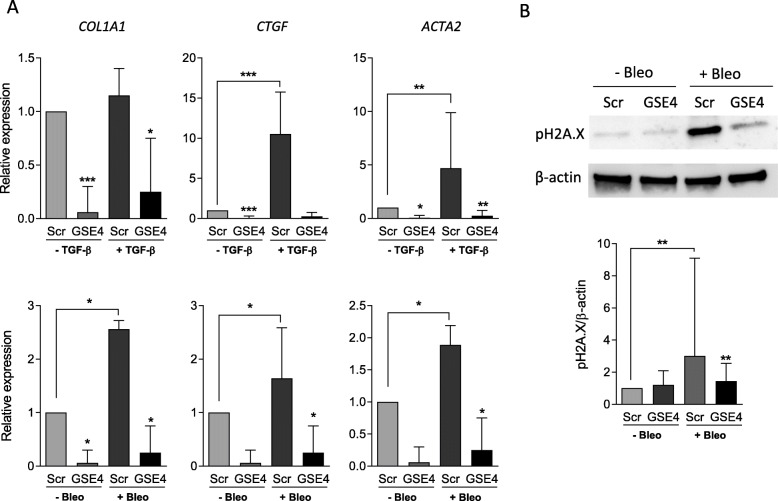
Effect of GSE4-lentiviral transduction to cultured dermal fibroblasts on profibrotic genes and phospho-H2A.X protein expression. Fibroblasts were transduced with scramble or GSE4 expression lentiviral vectors, and treated with TGF-β or bleomycin for 24 h as indicated. **A** Quantification of mRNA expression by quantitative RT-PCR of *COL1A1*, *CTGF* and *ACTA2* (*n* = 11)*.* (**B**) Representative image of Western blot analysis of phospho-H2A.X protein expression in a dermal fibroblast line (upper panel). Densitometric quantification shown as the ratio pH2A.X/β-actin (*n* = 10) (lower panel). *p < 0.05, **p < 0.01, ***p < 0.0001 (Median and IQR, Wilcoxon matched-pairs signed Rank Test)

By quantitative RT-PCR analysis, we found an up-regulation of *CTGF*, *ACTA2* and *COL1A1* gene expression in fibroblasts after TGF-β or bleomycin treatment. In both cases, a significant reduction of both the basal and induced expression of these genes was observed in GSE4-transduced compared to control peptide transduced fibroblasts (Fig. 3A). These data indicate that expression of the GSE4 peptide is able to inhibit both basal and TGF-β- or bleomycin-induced profibrotic gene expression in human dermal fibroblasts.

Bleomycin induced a significant increase in phospho-H2A.X, a robust marker of oxidative DNA-damage response [32], that was prevented by GSE4 but not scramble peptide expression in cultured dermal fibroblasts GSE4 (Fig. 3B).

## Discussion

Our data show a reduced age adjusted TL in SSc patients compared to controls. TS occurred in all disease subsets but it was more prominent in patients with lung involvement and in patients with anti-topoisomerase I antibodies, a serum marker also associated with lung involvement [33]. Previous studies have shown an association between TS and SSc lung disease but not in patients without lung disease [20, 21]. Adler et al. recently reported shorter TL in SSc patients with autoantibodies against TERF1 telomeric protein, a feature that they found associated with severe lung disease [22]. The clinical associations of this autoantibody specificity and the meaning of TS in that subset of SSc patients requires further confirmatory studies in different populations, since the prevalence of anti-telomeric autoantibodies seems low and highly variable in different ethnic groups.

We failed to detect correlations between TL and the duration or severity of skin or lung disease, suggesting that TS may be an early event and not a consequence of the progression of the disease. A similar observation has been reported in rheumatoid arthritis (RA), where TS was also independent of the duration and severity of the disease and associated with lung involvement [34]. In RA, TS was observed in both myeloid and lymphoid lineages, pointing to a hematopoietic stem cell defect [35].

In experimental lung fibrosis, TS and senescence of the epithelial cell component seem critical to the development of an abnormal reparative and fibrotic response, whereas the significance of TS in the stromal cell component is controversial [8, 10]. However, in other organs where epithelial damage is not involved, such as scleroderma skin, the role of fibroblast senescence in the pathogenesis of fibrosis can be more relevant [14, 16, 36]. Telomerase activity is not detected in adult fibroblasts and TL is dependent on cell divisions and oxidative stress mediated DNA damage [36, 37]. Telomere shortening or dysfunction leads to fibroblast cell senescence in models of oxidative damage and fibrosis [9, 12, 38, 39]. Oxidative stress is one of the major drivers of aging and cell senescence by inducing DNA damage response and telomere shortening or dysfunction, and it is also a pivotal factor in the pathogenesis of SSc where it is involved in autoimmunity, vascular damage and fibrogenesis [40–42]. Therefore, we investigated the effects of the GSE4 peptide which has shown to antagonize this process in different disease models [26–28].

We show that treatment with GSE4-nanoparticles ameliorates skin fibrosis in the bleomycin model, similarly to what has been observed in bleomycin induced lung fibrosis [26]. In cultured skin fibroblasts, GSE4 reduced the expression of relevant profibrotic genes, particularly in response to bleomycin, an agent able to induce oxidative stress and DNA damage. GSE4 actions include multiple protective effects on DNA damage and telomere shortening or dysfunction, particularly under oxidative stress, in other cellular models. We failed to demonstrate telomerase activity in cultured fibroblasts treated with GSE4 (data not shown) but confirmed a reduction in the DNA damage response as shown by a reduction of phospho-H2A.X. Therefore, targeting different processes leading to cell senescence is a potential strategy to reduce fibrosis in SSc [17].

Interestingly, GSE4 also reduced profibrotic TGF-β signaling in fibroblasts, an effect previously observed in alveolar epithelial cells [26]. Since TGF-β is not expected to induce DNA-damage or telomere dysfunction the mechanism is unclear. TGF-β signaling involves the production of mitochondrial ROS that promote Smad protein signaling, profibrotic gene expression and cell senescence [29, 30]. Since all selected profibrotic genes are downstream TGF-β signaling, it can be speculated that the observed effects of GSE4 on gene expression in unstimulated fibroblasts might relate to the signaling of autocrine TGF-β by ROS. GSE4 has been shown to increase the expression levels of the antioxidant genes SOD1, SOD2 and catalase thus decreasing oxidative stress in two different cellular models of disease [27, 28].

## Conclusions

Our results confirm an association between SSc and TS of peripheral blood leukocytes, and lower TL in the group of patients with lung involvement and ATA. Additionally, we have shown that targeting oxidative DNA damage with the dyskerin-derived GSE4 peptide reduces experimental skin fibrosis and profibrotic gene expression in human dermal fibroblasts. These findings suggest that processes leading to fibroblast senescence such as telomere attrition or DNA damage may be relevant pathogenic factors in the development of fibrosis in SSc and support the therapeutic potential of telomere or DNA protective strategies.

## Methods

### Telomere length analysis

TL was determined by Southern blot of DNA from 174 patients with SSc and 68 healthy age and sex-matched controls, from the same geographical area, and with a similar ethnic composition. Included SSc patients are part of a large prospectively characterized cohort with available whole blood DNA samples, representative of the unfractionated leukocyte pool. DNA from patients and controls had been collected along the same time-frame as part of previous genetic studies.

DNA was extracted by NZY gDNA isolation Kit (NZTech, Lisbon, Portugal) and its integrity checked by agarose gel electrophoresis. DNA was digested with 20 units/μl of the Hinf I/Rsa I restriction enzymes at 37 °C for 2 h. Telomere length was determined using the TeloTAGGG Telomere Length Assay (Roche Diagnostics, Mannheim, Germany) using 1,5 μg of genomic DNA [23]. The TL in the healthy control group was used to establish a TL standard curve and to obtain a z-score measure of the deviation TL of individual SSc patients from the age standardized curve. The z-score compared the TL value of each individual with the age-matched mean and standard deviation (SD) of the values obtained in the controls (individual’s value – population mean/population SD, age-matched population of within 9 years on average) [43]. Patient’s characteristics at the time of TL determination are depicted in Table 2.

**Table 2:** Demographic and clinical characteristics of SSc patients

Variables	SSc population (***n*** = 174)
Age (median, IQR)	54 (21)
Gender, female, n (%)	151 (86.8)
Race, Caucasian, n (%)	169 (97.1)
Smoking history, n (%)	74 (42.5)
Comorbidities, n (%)	
Diabetes mellitus	15 (8.6)
Hypertension	42 (24.1)
Dyslipidemia	40 (23.0)
MACE	11 (6.3)
Cancer	14 (8.1)
Disease duration^1^ median (IQR), years	7 (10)
Disease subtype, n (%)	
lcSSc	105 (60.3)
dcSSc	69 (39.6)
Autoantibodies, n (%)	
ATA	54 (31.0)
ACA	62 (35.6)
Clinical involvement of SSc, n (%)	
Pulmonary hypertension	17 (9.8)
Pulmonary (ILD)	57 (32.7)
Cardiac	29 (16.7)
Gastrointestinal	137 (78.7)
Renal	7 (4.0)
Previous Therapy, n (%)	
Glucocorticoids	98 (56.3)
Immunosuppressive drugs^2^	80 (46.0)
Maximal mRSS ever, median (IQR), range 0–51	10 (19)
Death by any cause, n (%)	34 (19.5)

^1^From first non-Raynaud disease manifestation. ^2^Azathioprine, Methotrexate, Cyclophosphamide, Mycophenolate mofetil, Rituximab or Leflunomide. (ILD) Interstitial lung disease. *lcSSc* limited cutaneous SSc, *dcSSc* diffuse cutaneous SSc, *ATA* anti-topoisomerase I antibodies, *ACA* anticentromere antibodies, *n* number, *mRSS* modified Rodnan skin score. Clinical definitions are described in Additional file 1 and according to reference [25]

### Mouse model of skin fibrosis

Dermal fibrosis was induced in 11 weeks old C3H/HeNCrl female mice (Charles River Laboratories; Saint Germain Nuelles, France) by daily subcutaneous injection of 100 μg of bleomycin (1 mg/ml; Mylan Pharmaceuticals, Barcelona, Spain) or 0.9% saline control into the shaved back skin for 4 weeks, as previously described [44].

To study the effect of GSE4 peptide in this model, 10 mice in each group were injected subcutaneously with 100 μl (5 μg) of GSE4- or scramble-containing nanoparticles every other day in the same area of bleomycin injection. Nanoparticles were produced as previously reported [45, 46].

### Histology

Skin was harvested and paraffin embedded for histological evaluation of the collagen dermal area by Masson’s trichrome staining (Sigma-Aldrich, St. Louis, USA). Masson’s stained full thickness skin sections were visualized on a Zeiss A1 microscope, and photographed and digitalized using an AxioCam ERc 5S camera and ZEN lite 2012 software (Zeiss, Jena, Germany). Collagen blue-stained fractional area was quantified using ImageJ software (http://rsb.info.nih.gov/ij).

### Transduction of human dermal fibroblasts with lentiviral vectors for GSE4 expression

Skin biopsies were obtained from involved forearm skin of 5 patients with SSc and 6 healthy individuals during minor skin surgery. Fibroblast cultures were established by explant growth in 10% fetal bovine serum/Dulbecco’s modified Eagle’s medium (DMEM) (Lonza, Viviers, Belgium) and used between passages 4 and 9. Fibroblasts maintained in DMEM with 0.5% fetal bovine serum, were stimulated for 24 h with 1 μg/ml bleomycin or 1 ng/ml of TGF-β1 (Calbiochem, Darnstadt, Germany).

Lentiviral particles were produced in HEK 293 T cells by transfecting the scramble control (pRRL-CMVIRES- EGFP) and GSE4-expressing (pRRL-CMV-GSE4-IRES-EGFP) vectors together with pCD-NL-BH and pMD.2G VSV. G packaging plasmids as previously described [47]. Forty hours after transfection, supernatants were recovered and used to transduce dermal fibroblast cells. Efficiency of transduction was monitored by flow cytometry and quantitative RT-PCR.

### Real-time quantitative RT-PCR

Total RNA from mouse skin biopsies and from human dermal fibroblasts was extracted using RNeasy Micro Kit (Qiagen, Copenhagen, Denmark) and TRI Reagent (Invitrogen) respectively, according to the manufacturer’s protocol. For the quantification of mRNA, 1 μg was used for first-strand complementary DNA synthesis with High Capacity cDNA Transcription Kit (Applied Biosystems, Foster City, CA, USA). Quantitative PCR (qPCR) analysis was carried out on a Roche LightCycler 480 II (Roche Diagnostics) instrument using Power Sybr Green PCR Master Mix (Applied Biosystems) according to the manufacturer’s recommendations. Primer sequences are listed in additional table (see Additional file 2). *Gapdh* (mouse) and *HPRT* (human) were used as endogenous reference. For relative quantification we compared the amount of target normalized to the endogenous reference using 2 − ΔΔCt formula (Ct = threshold cycle).

### Phospho-histone H2A.X Western blot analysis

Cell pellets were resuspended in lysis buffer (50 mM TRIS-HCl pH 8.0, 150 mM NaCl, 1% NP-40, 1% Na-Deoxycolate, 0.1% SDS, 2 mM EDTA pH 8.0) with a protease cocktail inhibitor (Roche Diagnostics). Protein concentration was determined by the DC method (Bio-Rad Laboratories, Hercules, CA, USA). Cell lysates (20 μg) were electrophoresed by SDS-PAGE and transferred to nitrocellulose membranes (Bio-Rad Laboratories). After blocking for 1 h with 10% BSA TBS-Tween, membranes were incubated overnight at 4 °C with anti-phospho-histone H2A.X (1/1000; #9718, Cell Signalling Massachusetts, USA). Secondary antibody was horseradish peroxidase-conjugated goat anti-rabbit IgG (1/2000; Invitrogen, G-21234, Carlsbad, CA, USA).

Values were normalized to β-actin levels detected by anti-β-actin HRP-conjugated antibody (1/25000; ab49900, Abcam). The protein bands were detected by chemiluminescence using ClarityTM Western ECL Substrate (Bio-Rad Laboratories, Hercules, CA, USA) in ImageQuantTM LAS 400 (GE Healthcare, Buckinghamshire, UK) and quantified by densitometry using ImageQuant TL (GE Healthcare).

### Statistical analyses

Data were analyzed using GraphPad Prism software v6.0 (GraphPad Software, San Diego, CA, USA). Quantitative data were analyzed by Mann-Whitney test or Wilcoxon matched-pairs signed Rank Test as appropriate. Correlation between TL and numerical variables was determined by Spearman rank test. Chi-square test was used to compare the frequency of severe TS (z-score < 10th percentile) in SSc and controls or between the different SSc subsets. All quantitative data are described as medians with interquartile range.

## Supplementary Information

**Additional file 1.** Clinical definitions included in Table 2.

**Additional file 2: Table S1.** Primer sequences used for quantitative real-time PCR analysis.

## Data Availability

Data generated or analyzed during this study are included in this published article and its supplementary information files. Data not shown are available from the corresponding author on reasonable request.

## Abbreviations

SSc: Systemic sclerosis
lcSSc: Limited cutaneous SSc
dcSSc: Diffuse cutaneous SSc
ILD: Idiopathic lung fibrosis
RA: Rheumatoid Arthritis
HC: Healthy Controls
MACE: Major adverse cardiovascular events
ATA: Anti-topoisomerase I antibodies
ACA: Anticentromere antibodies
n: Number
NS: Non significant
SD: Standard deviation
mRSS: Modified Rodnan skin score
TS: Telomere Shortening
TL: Telomere Length
COL1A1: Collagen type 1-alpha 1
ACTA2: α-smooth muscle actin
CTGF: Connective tissue growth factor
ROS: Reactive oxygen species
TSS: Total skin score
FVC: Forced vital capacity
DMEM: Dulbecco’s modified Eagle’s medium
qPCR: Quantitative PCR
