# Supplementary material for: Evidence of telomere attrition and a potential role for DNA damage in systemic sclerosis
Source: Immun Ageing. 2022 Jan 27;19:7. doi: 10.1186/s12979-022-00263-2 (PMC8793167; doi:10.1186/s12979-022-00263-2)
Supplement: Supplementary file 1 — Additional file 1. Clinical definitions included in Table 2. [file 12979_2022_263_MOESM1_ESM.pdf]

Additional file 1:

Clinical definitions[1]

- Interstitial lung disease (ILD), was diagnosed by high resolution computed tomography (CT) thoracic scan.

- Pulmonary arterial hypertension (PAH) was confirmed by right heart catheterization (RHC) and defined as a mean pulmonary artery pressure (mPAP) >25 mmHg with mean pulmonary wedge pressure <15 mmHg,

- Gastrointestinal involvement in SSc concerns the whole digestive tract. Esophageal involvement was defined as low esophageal sphincter or esophageal body dysfunction, diagnosed by manometry or gastroesophageal X-ray; intestinal involvement was clinically defined as significant diarrhea, constipation or sub-occlusive episodes.

- Cardiac manifestations include systolic dysfunction of left ventricle defined as ejection fraction < 55%, arrhythmias, pericarditis or conduction disorders.

- Renal crisis was defined either hypertensive (new-onset systemic hypertension plus either increased serum creatinine levels, proteinuria, hematuria, thrombocytopenia, or hemolysis) or normotensive (increased serum creatinine levels plus proteinuria, hematuria, thrombocytopenia, or hemolysis).

- Disease duration was defined as the time elapsed from first non-Raynaud's symptom to blood sample extraction and expressed in years.

- The presence of autoantibodies, anticentromeric B protein autoantibodies (ACA) and anti-topoisomerase-I (Scl70) was determined by Bioplex 2200 Multiplex Testing platform (Bio-Rad, CA, USA).

[1]Joven BE, Escribano P, Andreu JL, Loza E, Jimenez C, de Yebenes MJG, Ruiz-Cano MJ, Carmona L, Carreira PE. 2013 ACR/EULAR systemic sclerosis classification criteria in patients with associated pulmonary arterial hypertension. Semin Arthritis Rheum. 2018 Jun;47(6):870-876.
