# Supplementary material for: Evidence of telomere attrition and a potential role for DNA damage in systemic sclerosis
Source: Immun Ageing. 2022 Jan 27;19:7. doi: 10.1186/s12979-022-00263-2 (PMC8793167; doi:10.1186/s12979-022-00263-2)
Supplement: Supplementary file 2 — Additional file 2: Table S1. Primer sequences used for quantitative real-time PCR analysis. [file 12979_2022_263_MOESM2_ESM.pdf]

Additional File 2:

Table S1. Primer sequences used for quantitative real-time PCR analysis.

Gene Name	Forward Primer Sequence	Reverse Primer Sequence
Acta2 (mouse)	5´-GTCCCAGACATCAGGGAGTAA-3´	5´-TCGGATACTTCAGCGTCAGGA-3´
Ctgf (mouse)	5´- CTGCCTACCGACTGGAAG AC -3´	5´- TCGCATCATAGTTGGGTC TG -3´
Gapdh (mouse)	5´-CATGGCCTTCCGTGTTCCTA-3´	5´-GCGGCACGTCAGATCCA-3´
COL1A1	5´-GAGGGCCAAGACGAAGACATC-3´	5´-CAGATCACGTCATCGCACAAC-3´
ACTA2	5´-CTATGCCTCTGGACGCACAACT-3´	5´-CAGATCCAGACGCATGATGGCA-3´
CTGF	5´-GAGTGGGTGTGTGAC-3´	5´-GGCAGTTGGCTCTAA-3´
HPRT	5'-GCTGAGGATTTGGAAAGGGTG -3'	5'- CAGAGGGCTACAATGTGATGGC -3’

Acta2/ACTA2: actin alpha 2; Ctgf/CTGF: connective tissue growth factor; Gapdh: glyceraldehyde 3-phosphate dehydrogenase; COL1A1: collagen type I alpha 1 chain;; HPRT: hypoxanthine-guanine phosphoribosyltransferase.
